# Molecular and Functional Characterization of Trehalase in the Mosquito *Anopheles stephensi*

**DOI:** 10.3389/fphys.2020.575718

**Published:** 2020-11-19

**Authors:** Sanjay Tevatiya, Seena Kumari, Punita Sharma, Jyoti Rani, Charu Chauhan, Tanwee Das De, Kailash C. Pandey, Veena Pande, Rajnikant Dixit

**Affiliations:** ^1^Laboratory of Host-Parasite Interaction Studies, ICMR-National Institute of Malaria Research, New Delhi, India; ^2^Department of Biotechnology, Kumaun University, Nainital, India

**Keywords:** mosquito, migdut, trehalase (E.C 3.2.1.28), energy metabolism, reproduction, Plasmodium vivax

## Abstract

Like other insects, in blood-feeding mosquitoes, trehalase (TRE; EC 3.2.1.28), an enzyme that metabolizes trehalose, may influence a wide array of functions including flight, survival, reproduction, and vectorial capacity, but its role has not been investigated in detail. Here, we characterized a 1,839-bp-long transcript, encoding a 555-aa-long trehalase-2 homolog protein from the mosquito *Anopheles stephensi*. With a conserved insect homology, and in *silico* predicted membrane-bound protein, we tested whether trehalase (*As-TreH*) also plays a role in mosquito physiologies. Constitutive expression during aquatic development or adult mosquito tissues, and a consistent upregulation until 42 h of starvation, which was restored to basal levels after sugar supply, together indicated that *As-TreH* may have a key role in stress tolerance. A multifold enrichment in the midgut (*p* < 0.001819) and salivary glands (*p* < 4.37E-05) of the *Plasmodium vivax-*infected mosquitoes indicated that *As-TreH* may favor parasite development and survival in the mosquito host. However, surprisingly, after the blood meal, a consistent upregulation until 24 h in the fat body, and 48 h in the ovary, prompted to test its possible functional correlation in the reproductive physiology of the adult female mosquitoes. A functional knockdown by dsRNA-mediated silencing confers *As-TreH* ability to alter reproductive potential, causing a significant loss in the egg numbers (*p* < *0.001*), possibly by impairing energy metabolism in the developing oocytes. Conclusively, our data provide initial evidence that *As-TreH* regulates multiple physiologies and may serve as a suitable target for designing novel strategies for vector control.

## Graphical Abstract

Proposed functions of trehalase in mosquito tissues in response to altered pathophysiological conditions: Starvation, *Plasmodium vivax* infection, and reproduction.

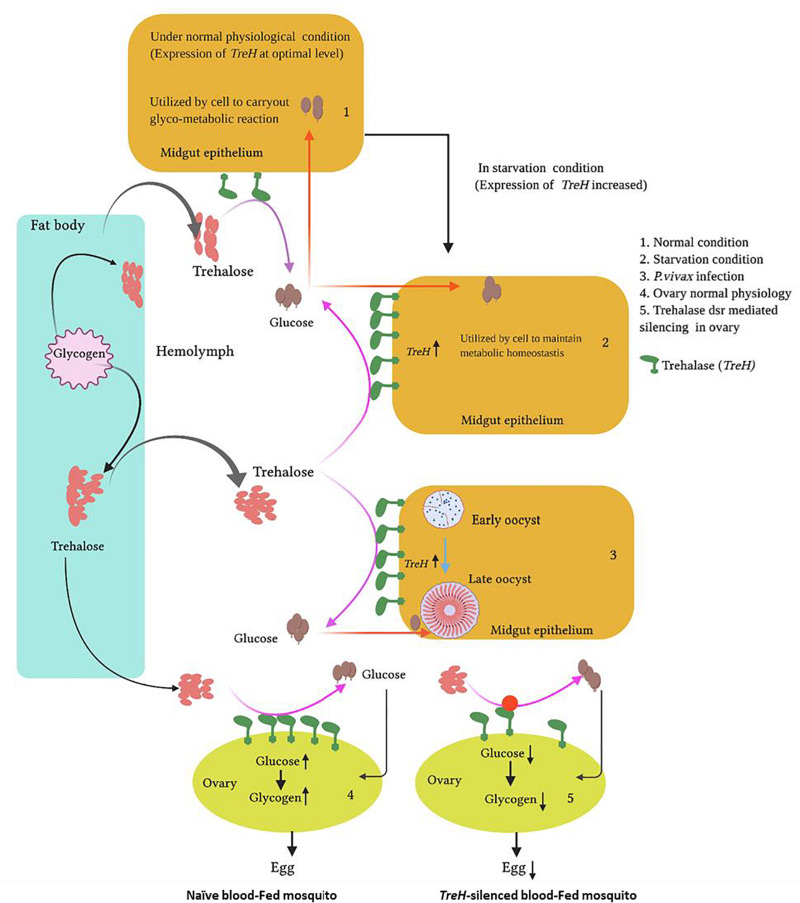

## Introduction

In nature, both adult male and female mosquitoes met their energy requirements from nectar sugar. However, adult female mosquitoes need a blood meal from a vertebrate host to nourish its eggs for reproductive success. Sugar metabolism in the adult mosquito influences the wide array of behavior including flight, survival, reproduction, and vectorial capacity. Like other insects, also in mosquitoes principal circulating sugar includes the non-reducing disaccharide trehalose (also known as hemolymph sugar), which is synthesized by the absorbed glucose in the fat body. However, trehalose exists in a wide variety of organisms, including nematodes, crustacean’s algae, plants, fungi, bacteria, and yeast, but lack in vertebrates. Trehalose performs multiple roles in these organisms, such as a structural component of cell walls in bacteria, fungi, and plants (Shimakata and Minatogawa, 2000; Müller et al., 2001; Wingler, 2002; Tang et al., 2018). In insects, it serves as a key source of energy to deal with abiotic stress and metabolic physiologies (McDougall and Steele, 1988; Shi Z. K. et al., 2017).

Structurally, trehalose is a disaccharide molecule consisting of two glucose units generated with the help of two enzymes, trehalose-6-phosphate synthase (TPS; EC 2.4.1.15) and trehalose-6-phosphate phosphatase (TPP; EC 3.1.3.12), in the fat body of the mosquitoes/insects. TPS catalyzes the formation of trehalose-6-phosphate (T-6-P) by transferring glucose from UDP-glucose to glucose-6-phosphate (Shukla et al., 2015). TPP then undergoes de-phosphorylation catalyzed by TPP, which removes the phosphate group from T-6-P to form trehalose, which is then released in the hemolymph for utilization (Shukla et al., 2016; Vanaporn et al., 2017).

Trehalase has been identified and characterized from bacteria to insects and shown to play an important role in desiccation and dehydration stress, thermal stress tolerance, asexual development, and virulence of insect mycopathogens (Shukla et al., 2016; Vanaporn et al., 2017; Hagan et al., 2018; Qiu et al., 2020). Trehalase has also been described as a potential virulence-associated gene in bacteria, as it is present in pathogenic bacteria but is missing in nonpathogenic bacteria (Shukla et al., 2016).

Insects encode two variants of the trehalase enzyme-soluble form (TRE-1) and membrane-bound form (TRE-2). Tre-1 is located inside the cell and hydrolyzes intracellular trehalose, whereas Tre-2 is a transmembrane enzyme facing the outside of the enzyme and hydrolyzing the extracellular trehalose (Chen et al., 2010; Zhao et al., 2016). In insects, the activity of trehalase varies widely over various stages of development and is highly affected by nutritional and environmental conditions such as modulation in activity of trehalase which plays a vital role in physiological adaptation to the cooling process in *Harmonia axyridis* (Shi Z. et al., 2016; Shi et al., 2019). Recently, Shukla et al. (2018) identify a new variant of trehalase from the insect oriental aquatic midge *Chironomus ramosus*.

The role of trehalase in energy metabolism under multiple physiologies has been investigated in various insects (Thompson, 2003; Mariano et al., 2009). Although identifiable in the genome, the direct function of trehalase has not been established in any mosquito species, until recent demonstration that RNA interference of trehalase causes reduced phenotypes associated with hydration level such as blood-feeding in *Culex pipiens* (Hagan et al., 2018). Earlier, knockdown of the trehalose transporter gene, *AgTreT1*, has been shown to affect stress adaptation and malaria pathogen development in the mosquito *An. gambiae* (Liu et al., 2013), while studies on RNA interference of the juvenile hormone III (JH) receptor methoprene-tolerant (Met) and 20E receptor, i.e., ecdysone receptor (EcR) suggested a regulatory switching role in the coordination of carbohydrate metabolism and female mosquito reproductive cycle maintenance, in the mosquito *Aedes aegypti* (Hou et al., 2015). Since trehalase is the only glucosidase that irreversibly hydrolyzes trehalose into glucose units to utilize it in cellular energy metabolism and that makes the trehalase enzyme an attractive molecular target, here we evaluated the transcriptional regulation of membrane-bound trehalase under distinct pathophysiological conditions in *Anopheles stephensi*, an important human malarial vector, in urban India. Using functional genomic approaches, we established a functional correlation of trehalase in reproduction, affecting egg-laying capacity (fertility) and survival of mosquitoes.

## Materials and Methods

### Mosquito Rearing and Maintenance

The Indian strain of mosquito *An. stephensi* was reared and maintained in the insectary at a temperature of 28 ± 2°C, with a relative humidity of ∼80% with a 12-h light–dark cycle, as mentioned previously (Sharma et al., 2015; Thomas et al., 2016). Adult mosquitoes were fed daily on sterile sugar solution (10%) using a cotton swab throughout the experiment. All protocols for rearing and mosquito infection to *Plasmodium vivax* were approved by the Institute Ethics Committee (ECR/NIMR/EC/2012/41).

### Sample Collection, RNA Extraction, and Infectivity Assay

Experimentally required tissues were dissected and pooled from the cold anesthetized adult female mosquitoes under different physiological conditions. To examine the tissue-specific expression of target genes, selected tissues such as hemocyte, spermatheca, and salivary gland and male reproductive tissues were dissected from 3- to 4-day-old naïve sugar-fed mosquitoes. Further, to check the effect of starvation on the mosquito survival and trehalase expression, we collected the midgut sample at different time points (24, 48, and 72 h) post starvation. To examine the molecular response under nutritional and reproductive physiological conditions, we collected ∼25 post blood-fed mosquito ovaries (4, 24, 48, and 72 h) and fat body (8, 16, 24, and 48 h). Midguts were dissected from 25 mosquitoes fed on rabbit blood and collected at different time points (3, 24, 48, 72 h, 10, and 14 days). For the collection of midgut infected with *P. vivax* sporozoites, 3–4-days-old *An. stephensi* mosquitoes were fed on the blood of *P. vivax*-infected patients (∼2% gametocytaemia) through a pre-optimized artificial membrane feeding assay as described earlier (Sharma, 2020). The confirmation of the *P. vivax* infection was done by staining the midgut with 5% mercurochrome to visualize the oocysts after 4 days of infection (DPI), as described earlier (Sharma, 2020). After confirmation, ∼20–25 mosquito tissues, i.e., midgut and salivary glands, were dissected at selected time points. Total RNA from the salivary gland, midgut, and other tissues was isolated using the standard Trizol method as described previously.

### cDNA Preparation and Gene Expression Analysis

Approximately 1 μg total RNA was utilized for the synthesis of first-strand cDNA synthesis using a mixture of oligo-dT and random hexamer primers and Superscript II reverse transcriptase as per the described protocol (Verso cDNA synthesis Kit, Cat#AB-1453/A, EU, Lithuania; Sharma et al., 2015; Thomas et al., 2016). For differential gene expression analysis, routine RT-PCR and agarose gel electrophoresis protocols were used. Approximately 25 ng of cDNA was used in 10-μL real-time PCR reactions. The relative abundance of the gene of interest was assessed using the SYBR Green qPCR master mix (Thermo Fisher Scientific), using the Bio-Rad CFX96 PCR machine. PCR cycle parameters involved an initial denaturation at 95°C for 15 min, 40 cycles of 10 s at 95°C, 15 s at 52°C, and 22 s at 72°C. After the final extension step, the melting curve was derived and examined for quality control. Each experiment was performed in three independent biological replicates. The relative quantification results were normalized with an internal control (actin) and analyzed by the 2^–ΔΔCt^ method and statistical analysis was performed using Origin 8.1 software. Differences between test samples and their respective controls were evaluated by the Student’s “*t*”*-*test.

### dsRNA-Mediated Gene Silencing Assays

For the knockdown of *As-TreH*, dsRNA primers carrying T7 overhang were synthesized as listed in Supplementary Table 2. The amplified PCR products were examined by agarose gel electrophoresis, purified (Thermo Fisher Scientific Gene JET PCR Purification Kit #K0701), quantified, and subjected to double-stranded RNA synthesis using Transcript Aid T7 high-yield transcription kit (Cat# K044, Ambion, United States), while the *dsrLacZ* gene of bacterial origin was used as a control. Approximately, ∼69 nl (3 μg/μL) of purified *dsRNA* product was injected into the thorax of a cold anesthetized 1–2-day-old female mosquito using a nano-injector (Drummond Scientific, CA, United States) as described earlier (Kumari et al., 2020). The silencing of the respective gene was confirmed by quantitative RT-PCR after 3–4 days of dsRNA injection.

### Starvation and Survival Assay

In the starvation assay, 2–3-day-old (∼230 adult female) mosquitoes were securely emerged in a cotton cage and kept in the insectary at the temperature of 28 ± 2°C and relative humidity of ∼80% with a 12 h light–dark cycle. The control mosquito group was kept on sugar meal (sugar+water), while the starved mosquito group was deprived of both sugar and water until the end of the experiment. All the test assays were performed in a non-crowded condition, and the number of living mosquitoes was tracked until all had died. In the survival time assay, the live mosquitoes were moved into new cages every alternate day, and the numbers of dead mosquitoes were counted daily basis. Gehan-Breslow-Wilcoxon test was used for statistical analysis of data using Prism 8.0 software (GraphPad Software Inc, CA).

### *In silico* Bioinformatic Analysis

Phylogenetic trees were prepared with selected trehalase protein sequences by the maximum likelihood (ML) method in MEGA X program as described previously (Kumar et al., 2018). We aligned all selected insects and other putative trehalase protein sequences using the ClustalW algorithm, where the reliability of the branching was tested by 1,000 bootstrap value of the replicates. The processed phylogenetic tree was examined based on clusters and nodes formed.

## Results

### Identification, Annotation, and Phylogenomics Analysis of *As-TreH*

**FIGURE 1:**
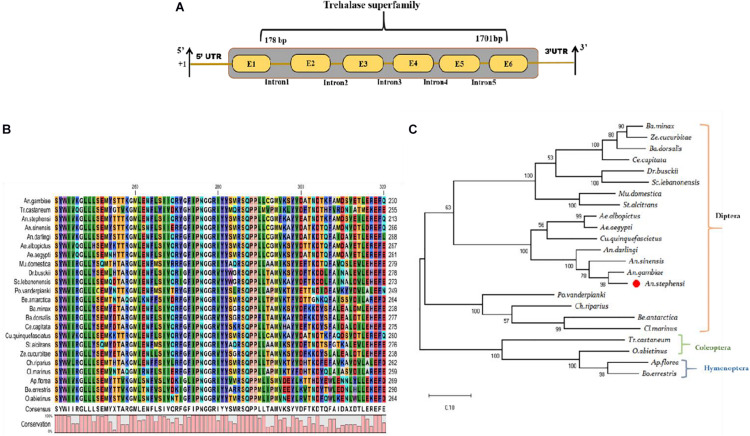
Genomic organization and molecular characterization of *An. stephensi As-TreH*: **(A)** Schematic representation of the genomic architecture of trehalase: Six brown color boxes (E1–E6) represent exons, and +1 indicates translation initiation site. A 50-bp UTR region is present on both the 5’ and 3’ ends of the transcript and one conserved trehalase superfamily domain present (1178–1701 bp). **(B)** Multiple-sequence alignment of mosquitoes and other insect-encoded trehalase homolog protein showing strong conservation among Anopheline mosquitoes. **(C)** Phylogenetic relationship of *As-TreH* proteins within the insect’s community. The red circle represents *Anopheles stephensi* in Diptera and the relationship of identified putative *As-TreH* with other Coleoptera, Hymenoptera showing a distance relationship with dipteran trehalase.

Our recent RNA-Seq study demonstrates that early (3–6-days) *P. vivax* infection significantly alters the metabolic machinery of the mosquitoes midgut (Tevatiya et al., 2019). Identification of a unique transcript, encoding the trehalase homolog protein, from the *P. vivax-*infected gut-RNAseq library, prompted further investigation of its possible role in mosquito physiology. The initial BLASTX analysis of the selected 1,839 bp long *TreH* transcript showed 91.61% identity with the *TreH* homolog of *Anopheles gambiae* (AGAP012053-PA), having a conserved trehalase domain (Figure 1A). The *AsTreH* transcript showed 53.2% identity with the *TreH* homolog of *Tribolium castaneum* (Coleoptera) and 52% identity with *Apis florea* (Hymenoptera) showed a less conserved region. Additionally, six copies of the trehalase gene in *Tribolium castaneum* (Coleoptera) and three copies of the trehalase gene in *Apis florea* (Hymenoptera) compared to only two copies in the Diptera reflect functional diversification of the trehalase enzyme during the course of evolution. For example, the existence of several copies of the trehalase gene in Coleoptera may be described as an adaptation to a trehalose-rich diet of insectivorous, detritivorous, and mycophagous species, as trehalose sugar is present in high concentrations in fungi (Nardelli et al., 2019).

A homology search against *An. stephensi* database further verified that the identified *As-TreH* is a 1,839 bp full-length (ASTEI06114-RA), a single-copy gene, which encodes a protein of 555 amino acids. Multiple-sequence alignment with other trehalase members, originating from Hymenoptera and Diptera orders, showed the highest identity and conservation with Anopheles mosquitoes (Figures 1B,C). Phylogenetic analysis showed the formation of two independent clades, where each clade defines a separate lineage for trehalase, which is further divided into specific subclades.

### Developmental and Tissue-Specific Transcriptional Response of *As-TreH*

**FIGURE 2:**
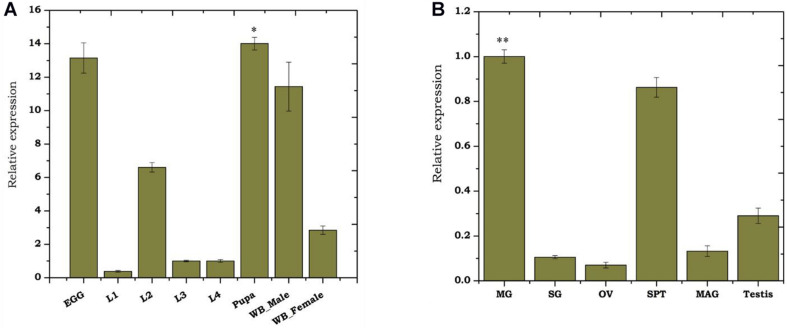
Transcriptional profiling of *As-TreH*: **(A)** Relative gene expression of *As-TreH* during developmental stages of *An. stephensi* mosquito i.e., L1 (instar larval stage 1), L2 (instar larval stage 2), L3 (instar larval stage 3), L4 (instar larval stage 4), pupa (*p* < 0.01716), and whole body in male (*p* < 0.01476) and female (*p* < 0.004148); the L3 stage was considered as controls for each test sample. **(B)** Tissue-specific expression kinetics of *As-TreH* in male and female mosquitoes. SG: salivary glands; MG: midgut (*p* < 0.000292); OV: ovary; SP: Spermatheca (*p* < 0.033); MAG: male accessory gland; testis. SG was considered as control for each test sample. Three independent biological replicates were considered for statistical analysis, viz. ^∗^*p* < 0.05; ^∗∗^*p* < 0.005; and ^∗∗∗^*p* < 0.0005, using the Student’s *t*-test.

To monitor the developmental regulation of trehalase, we performed real-time PCR-based transcriptional profiling. Though *As-TreH* showed constitutive expression throughout the aquatic developmental stages (Figure 2A), we observed a higher abundance during egg (active embryogenesis) and pupae stages (*p* < 0.01716) than other larval stages. We also observed that the relative expression of trehalase was higher (*p* < 0.01476) in adult males than in female mosquitoes (Figure 2A). Tissue-specific transcriptional profiling also showed a dominant expression of trehalase in the midgut (*p* < 0.000291), spermatheca (*p* < 0.033) of the adult female, and testis of adult male mosquitoes (Figure 2B). Together, we hypothesize that *As-TreH* may function in the maintenance of gut-nutritional homeostasis and reproductive physiology.

### *As-TreH* Influences Nutritional Homeostasis and Reproductive Physiology

**FIGURE 3:**
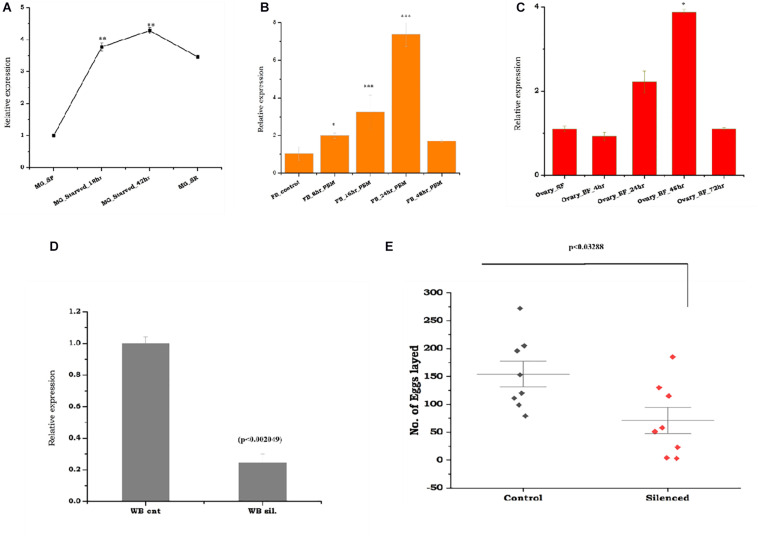
Comparative transcriptional profiling of the *As-TreH* genes in response to starvation, blood-feeding, and trehalase silencing effects on female fertility: **(A)** Temporal expression of mosquito trehalase in the starved mosquito midgut tissues (MG_starved 18 h (*p* < 0.000607), 42 h (*p* < 0.000239), and starved recovered mosquitoes (MG_SR/(*p* < 0.014342). **(B)** Relative expression profiling of *As-TreH* in a blood meal time-series experiment; the fat body (FB) was collected from naive sugar-fed adult female mosquitoes, and blood meal time-series expression is represented as 8 h (*p* < 2.06E-06), 16 h (*p* < 0.000388), 24 h (*p* < 6.03E-06), and 48 h. **(C)** Relative expression profiling of *As-TreH* in a blood meal time-series experiment; ovaries were collected from naive sugar-fed and blood-fed adult female mosquito, and the expression is represented as 4 h (*p* < 0.4253), 24 h (*p* < 0.07693), 48 h (*p* < 0.082802), and 72 h (*p* < 0.498118). **(D)** *As-TreH* silencing exhibited a >70% reduction in mRNA reduction as compared to control levels (*p* < 0.002049) tested in the whole body. Three independent biological replicates were considered for statistical analysis, viz. *p* < 0.05, *p* < 0.005, and *p* < 0.0005 using the Student’s *t*-test. **(E)** After mating with age-matched healthy male mosquitoes, *As-TreH-*silenced female mosquito lays reduced number of the eggs (*p* < 0.03288) than the control mosquito group. A minimum of three biological replicates was performed, and the females were allowed to lay eggs between the 3rd and 4th days (median with range; *n* = 3–5, 15–25 females/trial) and (*p* < 0.03288, Mann–Whitney *U*-test).

To test the above hypothesis, first, we evaluated the molecular correlation between sugar/water deprivation and mosquito survival. A 42-h-long deprivation of sugar and water not only caused significant mortality (*p* < 0.000239) but also elevated the expression of trehalase in surviving mosquitoes (Supplementary Table 1). However, rapid downregulation in response to re-supply of sugar indicated that *As-TreH* may have a role in the maintenance of nutritional physiology (Figure 3A). Next, to test the possible role in reproductive physiology, we examined the spatial and temporal expression of *As-TreH* in the midgut, fat body, and ovary of the blood-fed adult female mosquitoes. *As-TreH* showed a gradual enrichment until 24 h in the fat body (*p* < 6.03E-06) and 48 h in the ovary (*p* < 0.032802) (Figures 3B,C), indicating a unique role in egg development in the mosquito. Finally, to establish a functional correlation, we performed dsRNA-mediated gene silencing in virgin 3–4-day-old male as well as female mosquitoes. However, after silencing we did not observe any significant behavioral changes either in virgin or in mated mosquitoes (data not shown); surprisingly, we noticed a 50% reduction (*p* < 0.03288) in the egg-laying of the *As-TreH* silenced blood-fed female mosquitoes (Figures 3D,E), indicating that trehalase plays a key role in the regulation of mosquito reproductive physiology.

### *P. vivax* Infection Alters *As-TreH* Expression in the Midgut and Salivary Gland

**FIGURE 4:**
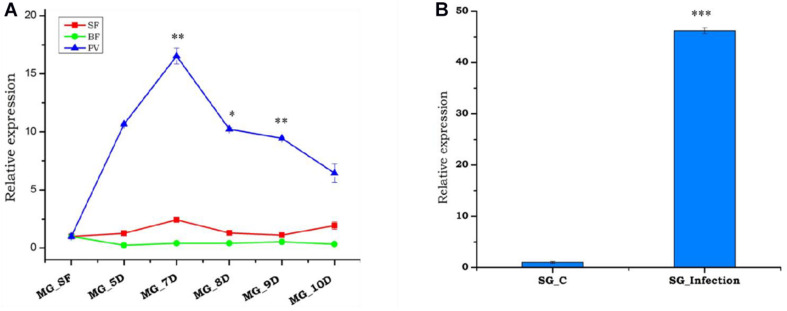
Relative expression profiling of trehalase under different conditions, i.e., sugar-fed, blood meal, and *Plasmodium vivax* infection in mosquito tissues. **(A)** Relative expression profiling of trehalase in a blood meal time-series experiment; midgut (MG) was collected from naive sugar-fed, blood-fed, and *P. vivax-*infected adult female mosquitoes, and significant modulation of expression in response to *P. vivax* is represented as time points 5D (*p* < 0.001148), 7D (*p* < 0.001819), 8D (0.00056), 9D (*p* < 0.002828), and 10D (*p* < 0.000289). D repentants number of days after blood-meal or *P. vivax* infection. **(B)** Transcriptional profiling of trehalase in response to post *P. vivax* infected (time point 12–14 D) SG glands (*p* < 4.37E-05) and age-matched normal blood-fed SG as a control. Three independent biological replicates were considered for statistical analysis, viz. ^∗^*p* < 0.05, ^∗∗^*p* < 0.005, and ^∗∗∗^*p* < 0.0005 using the Student’s *t*-test.

Previous studies have shown that the depletion of hemolymph trehalose sugar in *Plasmodium-*infected mosquitoes is likely due to rapid consumption by the fast-developing parasite, to meet their energy requirements (Mack and Vanderberg, 1978; Mack et al., 1979). Being a disaccharide, trehalose could not be utilized directly by the parasite, and also we retrieved a hexose transporter transcript of *Plasmodium* from RNAseq results; we opined that trehalase may have a role to convert trehalose to a sugar molecule. To test this, we tracked the trehalase expression during the development of oocyst in the gut, and sporozoite invaded salivary glands of *P. vivax-*infected female mosquitoes. A multifold enriched expression during early to late-stage oocysts in the gut as well as salivary glands (Figures 4A,B) suggests that trehalase may significantly contribute to hydrolyzes of the trehalose to provide glucose for the rapid proliferation of parasites and generation of new biomass.

## Discussion

Here, we demonstrated that insect homolog trehalase may play a significant role in maintaining the nutritional homeostasis and reproductive physiology of the mosquito tissues. We characterized an 1,839-bp-long unique transcript, encoding 555-aa-long trehalase*-2*, originally identified from the *P. vivax-*infected gut and salivary gland RNAseq database of mosquito *An. stephensi* (Tevatiya et al., 2019). We noticed that putative *TreH* is a membrane-bound protein, with conservation among insects and mosquito species, and may have a similar function in nutritional physiology (Shukla et al., 2015). Previous studies suggest that except vertebrate trehalase, which is required for metabolization of ingested trehalose, in other animals including insects, trehalase is involved in the recovery from diverse stress conditions, and regulation of energy metabolism and nutritional physiological homeostasis (Shukla et al., 2016; Shi J.F. et al., 2017; Vanaporn et al., 2017; Karpova et al., 2019).

Our observation of an enriched expression of *As-TerH* in developing eggs and pupae correlates its active role in the trehalose metabolization, to fulfill the nutritional sugar supply demand for highly metamorphic development stages (Gu et al., 2009; Shi J.F. et al., 2016). Consistent with previous studies, an abundant expression in the age-matched adult male than in female mosquitoes indicated a hyper-metabolic energy status of males, which may likely meet sexual behavioral activities (Hou et al., 2015). However, we also observed that sugar and water deprivation for a longer duration (∼42 h) reduces the survival rate of the mosquitoes, but a parallel upregulation of trehalase transcript after starvation, and restoration upon sugar re-supplementation, in the gut of surviving mosquitoes further corroborate previous studies that trehalose metabolization is crucial to stress tolerance and nutritional homeostasis maintenance (Liu et al., 2013). In their recent study, Hou et al. (2015) showed a dynamic expression regulation of trehalose metabolizing enzymes TPS, TPP, and trehalase transcripts, i.e., post eclosion, a sharp downregulation, and increased expression in the mosquito *Ae. Aegypti* within 36 h after blood-feeding (Hou et al., 2015). A direct functional role for any of these enzymes is yet to unravel; however, RNA interference studies have demonstrated that JH and 20E are key to regulating the coordination of carbohydrate metabolism during the mosquito reproduction cycle (Hou et al., 2015). 20E, JH, and insulin-like peptide (ILP) signaling pathways also seem to regulate the transcription of trehalase genes in the *Colorado potato* beetle (Shi Z. K. et al., 2017). We observed that blood-feeding boosts the gradual upregulation of trehalase until 24 h in the fat body and 48 h in the ovary; we tested whether trehalase influences the reproductive physiology. Functional knockdown experiments showed a 50% reduction of eggs laid by the trehalase gene silenced mosquitoes, than the control mosquito group. As oocytes synthesize and accumulate glycogen, trehalose from the hemolymph could be the main source for this synthesis; our data strengthen the hypothesis that a membrane-bound trehalase may play a crucial role in meeting the high nutritional requirement for oocytes development (Santos et al., 2008; Kuang et al., 2018).

Emerging studies have also demonstrated that *Plasmodium* infection imbalances glucose/trehalose metabolism, where gut microbiota likely plays a key role in the regulation of malaria parasite development in the mosquitoes (Wang et al., 2020). After traversing the midgut, a single ookinete with no reserved food (glycogen or lipid) transforms into an oocyst that produces thousands of haploid sporozoites. The parasite must scavenge nutrients from the host. Owing to the membrane-bound nature, we tested how fast-developing *P. vivax* oocysts or sporozoites influence trehalase expression in the gut or salivary glands of mosquitoes. We noticed that *P. vivax* infection may severely affect the energy metabolism of its vector, by modulating the trehalase expression in both tissues. Since the molecular nature of salivary sporozoites is different from circulatory sporozoites in the hemolymph (Tevatiya et al., 2019) and only attains its maturity or virulence through high energy requirements from its host, the tissue-specific altered expression of trehalase may likely favor *Plasmodium* development, survival, or transmission.

## Conclusion

In summary, we provide evidence that insect homolog membrane-bound trehalase plays a crucial role in the maintenance of nutritional homeostasis and reproductive physiology of the mosquito *An. stephensi*. An altered expression in response to *P. vivax* infection further supports the hypothesis that sugar metabolism-related enzymes, such as trehalase, may serve as a unique target for the design of vector control strategy.

## Data Availability Statement

All datasets generated for this study are included in the article/Supplementary Material.

## Ethics Statement

The studies involving human participants were reviewed and approved by the ECR/NIMR/EC/2012/41. The patients/participants provided their written informed consent to participate in this study.

## Author Contributions

ST and RD conceived the scientific hypothesis and designed the experiments. SK, CC, JR, TD, and PS were responsible for the technical support for tissue dissection, collection, and gene profiling, data review, and presentation. RD and KP contributed to the reagents, materials, and analysis tools, and edited the manuscript. All authors read and approved the final manuscript.

## Conflict of Interest

The authors declare that the research was conducted in the absence of any commercial or financial relationships that could be construed as a potential conflict of interest.
