# Supplementary material for: Molecular and Functional Characterization of Trehalase in the Mosquito Anopheles stephensi
Source: Front Physiol. 2020 Nov 19;11:575718. doi: 10.3389/fphys.2020.575718 (PMC7710876; doi:10.3389/fphys.2020.575718)
Supplement: Supplementary file 1 [file Data_Sheet_1.PDF]

Supplementary data

Molecular and functional characterization of Trehalase in the mosquito *Anopheles stephensi*

Sanjay Tevatiya[1], Seena Kumari[1], Punita Sharma[1], Jyoti Rani[1], Charu Chauhan[1], Tanwee Das De, Kailash C Pandey[1], Veena Pande[2], Rajnikant Dixit[1]*

1. Laboratory of Host-Parasite Interaction Studies, ICMR-National Institute of Malaria Research, Dwarka, New Delhi-110077, India
2. Department of Biotechnology, Kumaun University, Nainital, Uttarakhand, India

[*]**Correspondence:** Rajnikant Dixit**; Email:** dixitrk@mrcindia.org

Table-1: Time-dependent Starvation effect on adult female mosquito mortality. A total of 230, three days old naïve adult female mosquitoes were kept on starvation, while an equal number of naïve female mosquitoes were kept as unstarved. Values represent an average of three independent replicates.

Time (after sugar/water removal)	Starved (deceased)	Control (deceased)
After 12 hr	-7	-3
After 24 hr	-19	-6
After 36 hr	-35	-4
After 48 hr	-53	-8
After 60 hr	-27	-5
After 72hr	-89	-6

After 72hrs, all the starved mosquitoes died while in control 32 mosquitoes died.

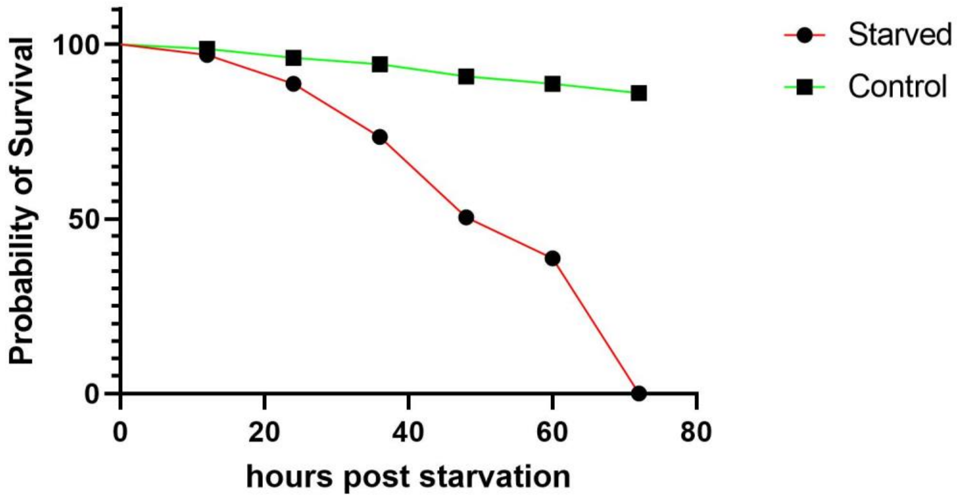

Figure 1:-Starvation effect and the survival curve analysis of the mosquito *An. stephensi* ($p < 0.0001$, Gehan-Breslow-Wilcoxon test)

Table-2: Primer list

**S.No.**	**Gene name**	**Primer sequences**
	Actin	Fw: 5’ TGCGTGACATCAAGGAGAAG 3’<br>Rev: 5’GATTCCATACCCAGGAACGA 3’
	DSR_Lacz*	Fw: 5' TAATACGACTCACTATAGGGGAGTCAGTGAGCGAGGAAG 3’<br>Rev: 5’ TAATACGACTCACTATAGGGTATCCGCTCACAATTCCACA 3’
	Trehalase_DSR *	Fw: 5' TAATACGACTCACTATAGGGGGATTCGTACTGGATCGTAA 3’<br>Rev: 5' TAATACGACTCACTATAGGGAGCTGATGATTGTTCACCTC 3’
	Trehalase	Fw: 5' ACGTTTAACAAAACGAGAGC 3’<br>Rev: 5' GCCGGTATTGATGGAGTAT 3’

***Red color sequence represents the T7 overhang**
